# 6′-*O*-Galloylpaeoniflorin Attenuates Cerebral Ischemia Reperfusion-Induced Neuroinflammation and Oxidative Stress via PI3K/Akt/Nrf2 Activation

**DOI:** 10.1155/2018/8678267

**Published:** 2018-03-25

**Authors:** Zhongmei Wen, Weichen Hou, Wei Wu, Yang Zhao, Xuechao Dong, Xiaoxue Bai, Liping Peng, Lei Song

**Affiliations:** ^1^Department of Respiratory Medicine, The First Hospital of Jilin University, 71 Xinmin Street, Changchun, China; ^2^Department of Neurology, The First Hospital of Jilin University, 71 Xinmin Street, Changchun, China; ^3^Department of Neurosurgery, The First Hospital of Jilin University, 71 Xinmin Street, Changchun, China; ^4^Cadre's Ward, The First Hospital of Jilin University, 71 Xinmin Street, Changchun, China

## Abstract

6′-*O*-galloylpaeoniflorin (GPF), a galloylated derivative of paeoniflorin isolated from peony root, has been proven to possess antioxidant potential. In this present study, we revealed that GPF treatment exerted significant neuroprotection of PC12 cells following OGD, as evidenced by a reduction of oxidative stress, inflammatory response, cellular injury, and apoptosis in vitro. Furthermore, treatment with GPF increased the levels of phosphorylated Akt (p-Akt) and nuclear factor-erythroid 2-related factor 2 (Nrf2), as well as promoted Nrf2 translocation in PC12 cells, which could be inhibited by Ly294002, an inhibitor of phosphoinositide 3-kinase (PI3K). In addition, Nrf2 knockdown or Ly294002 treatment significantly attenuated the antioxidant, anti-inflammatory, and antiapoptotic activities of GPF *in vitro*. *In vivo* studies indicated that GPF treatment significantly reduced infarct volume and improved neurological deficits in rats subjected to CIRI, as well as decreased oxidative stress, inflammation, and apoptosis, which could be inhibited by administration of Ly294002. In conclusion, these results revealed that GPF possesses neuroprotective effects against oxidative stress, inflammation, and apoptosis after ischemia-reperfusion insult via activation of the PI3K/Akt/Nrf2 pathway.

## 1. Introduction

Stroke, a common neurological disease, has become an important public health problem worldwide. In 2013, stroke was the second most common cause of deaths (11.8% of all deaths) worldwide, and the third most common cause of disability (4.5% of disability-adjusted life years from all cause) [1]. Statistical data indicated that ischemic strokes account for approximately 87% of all stroke cases [2]. With the emergence of an aging population, the incidence of ischemic stroke is increasing every year. The general therapeutic strategy for ischemic stroke is to restore the blood flow in the infarct area. However, human intervention or spontaneous reperfusion may further aggravate the nerve injury and neurological dysfunction caused by ischemia, which is known as cerebral ischemia-reperfusion injury (CIRI) [3]. The mechanism underlying CIRI has not yet been fully clarified. Recent studies indicate that after reperfusion, oxidative stress caused by a large amount of free radical production coupled with a decrease in scavenging capacity is the pathological core of CIRI [3, 4]. Excessive oxidative stress causes lipid peroxidation of nerve cell membranes and organelles, protein nitration, destruction of nucleic acids, and severe inflammatory reactions. Nevertheless, there is still no effective treatment available to protect against the oxidative stress and inflammatory response caused by CIRI.

Nuclear factor-erythroid 2-related factor 2 (Nrf2) is a pivotal transcription factor that maintains cellular redox homeostasis. Under physiological conditions, Nrf2 locates in the cytoplasm as an inactive form binding to its inhibitor kelch-like ECH-associated protein 1 (Keap1). When oxidative stress occurs, Nrf2 dissociates from Keap1, translocates into the nucleus, and binds to the antioxidant response element (ARE) to promote the gene expression of detoxifying enzymes and antioxidant enzymes [5]. Nrf2 plays a crucial role in maintaining normal physiological processes in the brain. Nrf2 knockout in mice results in proteasome dysfunction, neuronal apoptosis, age-related forebrain atrophy, and neurobehavioral deficits [6], as well as increased levels of dopamine and 5-serotonin metabolites in the brain [7]. Nrf2 has been reported to play a neuroprotective role after CIRI. Its expression significantly increases at 2 h and peaks at 8 h after CIRI in the infarct area [8]. Compared with wild-type mice, significant increases of the infarct volume and neurological function score are found in Nrf2 knockout mice after CIRI [9, 10]. In addition, the administration of Nrf2 agonist *tert*-butylhydroquinone or some herbal ingredients, such as protocatechuic aldehyde, genistein, or sulforaphene, can improve motor function and reduce the infarction volume in a murine model of CIRI [10–13]. These studies indicate that Nrf2-targeting drugs are capable of reducing CIRI and therefore could be used for the treatment of ischemic stroke.

6′-*O*-Galloylpaeoniflorin (GPF), a galloylated derivative of paeoniflorin isolated from peony root, is composed of d-glucose with galloyl and benzoyl substituents [14]. GPF has been shown to have a significant antioxidant capacity as it can scavenge reactive oxygen species (ROS) induced by ultraviolet B radiation or H_2_O_2_ in human keratinocytes, reduce oxidative stress-induced proteins, lipids, and DNA damage, and inhibit the mitochondrial pathway of apoptosis [15, 16]. Matsuda et al. [17] have found that the galloyl group is a necessary part of GPF for scavenging free radicals and that the ability of GPF to scavenge 1,1-diphenyl-2-picrylhydrazyl free radicals was stronger than that of alpha-tocopherol. However, whether GPF exerts a neuroprotective effect against CIRI by modulating oxidative stress still remains unclear. In this study, we aimed to determine the neuroprotective effects of GPF. The antioxidative stress, anti-inflammatory, and antiapoptosis activities of GPF were detected in the oxygen-glucose deprivation-induced PC12 cells and rat CIRI model in multidetecting systems. In addition, the signaling pathway was investigated. The results of the study provided evidence to support that GPF has potential value to develop a new drug for ischemic stroke treatment.

## 2. Materials and Methods

### 2.1. Cell Culture and Treatment

The rat pheochromocytoma cell line, PC12 cells (China Cell Collection Center, Beijing, China), was incubated in a 5% CO_2_ incubator at 37°C with Dulbecco's Modified Eagle medium (Gibco, Carlsbad, CA, USA) containing 10% fetal bovine serum (Gibco), 100 U/mL penicillin and 100 *μ*g/mL streptomycin. The culture medium was replaced every three days.

For oxygen-glucose deprivation (OGD) model establishment, PC12 cells were incubated with Earle's Balanced Salt Solution (Sigma-Aldrich, St. Louis, MO, USA) without glucose in a 1% oxygen-supplying incubator for 4 h. The cells were then transferred into an incubator at normal conditions for another 24 h.

To determine the antioxidant, anti-inflammatory, and neuroprotective effects of GPF (Sigma-Aldrich), PC12 cells were treated with different doses of GPF (0, 10, 50, and 100 *μ*M, resp.) for 1 h and then subjected to OGD. For PI3K/Akt inhibition *in vitro*, the PI3K inhibitor Ly294002 (Sigma-Aldrich) was added to the culture medium at a final concentration of 50 *μ*M. For Nrf2 knockdown, PC12 cells were transfected with 50 nM Nrf2 siRNA or scramble RNA using Lipofectamine 2000 (Thermo Fisher Scientific, Waltham, MA, USA).

### 2.2. Animals and Treatment

Healthy male Wistar rats, 7-8 months old and 270 ± 10 g weight, were provided by the Animal Laboratory of Jilin University. All protocols were performed in accordance with Guidance for the Care and Use of Laboratory Animals, formulated by the National Institutes for Health Research. The experiments were approved by the Ethics Committee of Jilin University.

For GPF treatment, rats were intraperitoneally injected with 2.5, 5, or 10 mg/kg GPF daily, respectively, for 14 days until establishment of the CIRI model. In order to inhibit the PI3K/Akt pathway *in vivo*, Ly294002 was injected into the lateral ventricle (20 mM, 5 *μ*L, from the bregma: anteroposterior, −0.8 mm; lateral, 1.5 mm; depth, 3.5 mm) daily for 7 days before CIRI model establishment.

### 2.3. Establishment of the CIRI Model

The rats were subcutaneously anesthetized with ketamine (100 mg/mL, 100 mg/kg), xylazine (20 mg/mL, 2.5 mg/kg), and acepromazine (2.5 mg/mL, 2.5 mg/kg) (Jinan Wanxingda Chemical Co., Ltd., Jinan, China). The rats were fixed on an operating board in the supine position. After disinfection, a median incision was made on the neck. The left common carotid artery, external carotid artery, and internal carotid artery were dissociated, in order. A small cut was made on the proximal end of the common carotid artery using eye scissors. A monofilament nylon suture (Beijing Sunbio Biotech Co., Ltd., Beijing, China) with a diameter of 0.26 mm was inserted, reaching the beginning of the middle cerebral artery through the internal carotid artery. Then, the blood flow of the left middle cerebral artery was blocked for 2 h by tightening up the nylon suture. After regaining consciousness, the rats were housed at 20–25°C and allowed free access to food and water. In the sham group, the rats received the same surgical procedures except for arteries occlusion.

### 2.4. Cell Counting Kit-8 (CCK-8) Assay

The viability of PC12 cells was determined using the CCK-8 assay (Sigma-Aldrich). Briefly, 3000 PC12 cells were plated in each well of a 96-well plate. 100 *μ*L medium was added to each well. At 24 h after incubation, 10 *μ*L of the CCK-8 solution was added to each well, and the plate was incubated for another 2 h. The optical density of each well was measured at 450 nm using a microplate reader (BioTek, Winooski, VT, USA).

### 2.5. Evaluation of Infarct Volume

At 24 h after reperfusion, the rats were sacrificed and the whole brains were rapidly removed. Coronal sections were cut into 2 mm slices and stained with 2% 2,3,5-triphenyltetrazolium chloride (TTC, Sigma-Aldrich) for 10 min at 37°C, followed by immersion in 4% paraformaldehyde overnight. The infarct volume was measured using microscope image-analysis software (Image-Proplus, Rockville, MD, USA) according to the following formula: [contralateral hemisphere area − (ipsilateral hemisphere area − infarct area)/contralateral hemisphere area] × 100%.

### 2.6. Real-Time Quantitative Polymerase Chain Reaction (qPCR)

In accordance with the manufacturer's instructions, total RNA was extracted with TRIzol reagent (Invitrogen, CA, USA). cDNA was synthesized using a Superscript III Reverse Transcriptase kit (Invitrogen). A solution of 2.5 *μ*L of cDNA and 1 *μ*L of specific primers was thoroughly mixed with TransStartTM SYBR Green qPCR Supermix (Transgene, Beijing, China). qPCR was performed using an ABI 7500 PCR system (ABI, USA). GAPDH mRNA served as an internal reference. The PCR products were quantitatively analyzed. The primer sequences were used in accordance with the previous studies [18, 19]: TNF-*α* upstream primer, 5′-TCTCAAAACTCGAGTGACAAG-3′, downstream primer, 5′-AGTTGGTTGTCTTTGAGATCC-3′; IL-1*β* upstream primer, 5′-AACTGTCCCTGAAC-TCAACTG-3′, downstream primer, 5′- TGGGAACATCACACACTAGC-3′; GAPDH upstream primer, 5′-AAATTCAACGGCACAGTCAA-3′, and downstream primer, 5′-GTCTTCTGGGTGGCAGTGAT-3′.

### 2.7. Western Blot

Western blot was used to determine Nrf2, Akt, phosphorylated Akt (p-Akt), and heme oxygenase 1 (HO-1) protein expression. Briefly, total or nuclear protein was extracted using RIPA lysate (Cell Signaling Technology, Boston, MA, USA) or CelLytic™ NuCLEAR™ Extraction Kit (Sigma-Aldrich), respectively, according to the manufacturer's protocol. Protein concentrations were determined with the Bicinchoninic Acid Protein Kit (Cell Signaling Technology). Total proteins (15 *μ*g) of each sample were mixed with 4 *μ*L of 6x loading buffer, loaded on a discontinuous 10% sodium dodecyl sulfate-polyacrylamide gel, separated by electrophoresis, and transferred onto a PVDF membrane (Thermo Fisher Scientific) by the wet method. The membrane was blocked with bovine serum albumin (Boster, Wuhan, China) and skimmed milk for 2 h, washed with tris-buffered saline containing Tween 20 (TBST; Boster), and incubated with the primary antibody rabbit anti-polyclonal antibody IgG (1 : 1000; Abcam, Hong kong, China) at 4°C overnight. After washing with TBST, the membrane was incubated with the horseradish peroxidase-labeled secondary antibody goat anti-rabbit polyclonal antibody IgM (Abcam) at room temperature for 2 h, followed by detection with the enhanced chemiluminescence reagent.

### 2.8. Oxidative Stress Determination

The cellular ROS level was evaluated using a DCFH-DA kit (Invitrogen, Carlsbad, CA, USA). Briefly, 2 × 10^5^ PC12 cells were seeded into 6-well plates. In accordance with the instructions, a final concentration of 20 nM DCFH-DA was added to the cells for 30 min in an incubator containing 5% CO_2_ and 90% humidity. The positive stained cells (green fluorescence) were observed and photographed under a fluorescence microscope (Olympus, Tokyo, Japan).

### 2.9. Mitochondrial Membrane Potential (MMP)

PC12 cells (2 × 10^5^/mL) were seeded into 6-well plates, followed by treatment with dimethyl sulfoxide-dissolved rhodamine 123 (Invitrogen), 5 *μ*M Fluo-3/AM Ester, and 0.0625% Pluronic F127 (proportion by weight) in an incubator with 5% CO_2_ and 90% humidity for 45 min. After washing twice with PBS, the treated cells were centrifuged at 1500 rpm for 5 min. The mean fluorescence intensity of the cells in each group was measured at 485 nm with a flow cytometer.

### 2.10. Biochemical Assessment

All the processes were strict in accordance with the instructions of the superoxide dismutase (SOD) and glutathione peroxidase (GSH-Px) activity assay kits (Jiancheng, Nanjing, China), as well as the glutathione (GSH) and malondialdehyde (MDA) level measurement kits (Jiancheng). The disrupted cell or tissue lysate was centrifuged at 12000*g* for 5 min, and the supernatant was mixed with detection solution and incubated for 40 min at 95°C in a water bath. After cooling, the samples were centrifuged at 4000*g* for 10 min. The optical density values of each group were measured and recorded at 532 nm with a 1 cm light path. The SOD and GSH-Px activities as well as the GSH and MDA levels were calculated based on the optical density values.

### 2.11. Lactate Dehydrogenase (LDH) Activity

The OGD-induced cellular damage was evaluated by lactate dehydrogenase (LDH) activity detection. Briefly, 20 *μ*L of cell culture supernatant was mixed with 25 *μ*L of detection buffer (Jiancheng) and 5 *μ*L of coenzyme I (Jiancheng) at 37°C for 15 min, then 25 *μ*L of 2′,4′-dinitrophenylhydrazine (Jiancheng) was added and allowed to react for 15 min under the same conditions. Finally, 250 *μ*L of 0.4 M NaOH was added, and the mixture was incubated at room temperature for 5 min. The absorbance value of each group was measured at 450 nm with a microplate reader (BioTek, Beijing, China).

### 2.12. Enzyme-Linked Immunosorbent Assay (ELISA)

The TNF-*α* and IL-1*β* levels in the cell culture medium were evaluated with ELISA. In brief, PC12 cells were treated with 500 *μ*L of ice-cold carbonate buffer (100 mM Na_2_CO_3_, 50 mM NaCl, pH 11.5) with protease inhibitors, and then the cells were disintegrated with an ultrasonic homogenizer. The mixture was centrifuged at 12000*g* for 45 min. The supernatant was obtained, and the caspase-3 content was measured in accordance with the kit instructions (Abcam).

### 2.13. Terminal Deoxynucleotidyl Transferase dUTP Nick End Labeling (TUNEL) Assay

The apoptotic cells were stained with TUNEL reagents. Briefly, the cells were fixed with 4% paraformaldehyde at 25°C for 20 min, washed three times with PBS, treated with 1% Triton X-100, and blocked with 3% H_2_O_2_ at room temperature for 10 min. After washing with PBS three times, each sample was reacted with TdT enzyme reaction buffer containing TRITC-5-dUTP and TdT Enzyme (Coolrun, Shenzhen, China) at 37°C in dark for 60 min and washed three times with PBS. Nuclei were visualized with DAPI staining. The TUNEL-stained cells were observed under a fluorescence microscope.

### 2.14. Histological Analysis

Histological analysis was performed on the brain sections to detect the expression of ionized calcium-binding adapter molecule 1 (Iba1), phosphorylated p38 (p-p38), and phosphorylated JNK (p-JNK) as well as apoptotic cells. Briefly, formalin and paraffin were used to fix and embed the tissues, respectively. The samples were heated to retrieve the antigen. Antibodies recognizing the involved target proteins (Abcam) were employed to stain these proteins, followed by counterstaining of the nuclei with hematoxylin. To detect apoptosis *in vivo*, TUNEL staining was conducted using an In Situ Cell Death Detection Kit (Roche, Germany), according to the manufacturer's procedures.

### 2.15. Statistical Analysis

Data were analyzed using SPSS 17.0 software for Windows (SPSS Inc., Chicago, IL, USA). Measurement data were expressed as the mean ± standard deviation. Grouped data were expressed as a relative number. Group comparison was carried out using analysis of variance and the Q test. The significance level was *α* = 0.05.

## 3. Results

### 3.1. GPF Lessens OGD-Induced Oxidative Stress and Inflammatory Reactions

**Figure 1:**
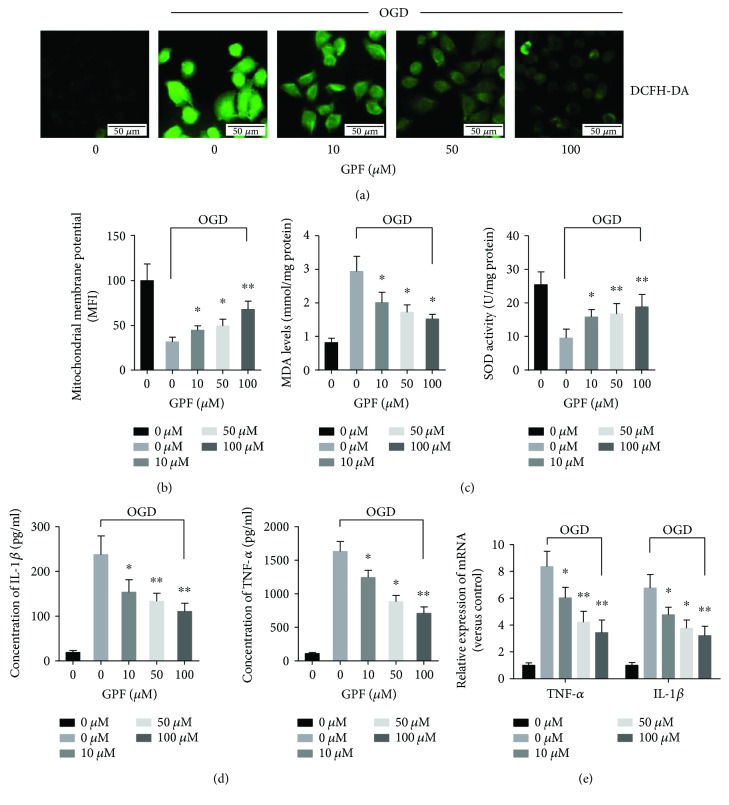
The antioxidative and anti-inflammatory activities of GPF in the OGD model. To determine the antioxidant and anti-inflammatory effects of GPF, PC12 cells were pretreated with different doses of GPF (0, 10, 50, or 100 *μ*M, resp.) for 1 h before subjected to OGD. PC12 cells without GPF and OGD treatment were set as a control. Typical representative DCFH-DA staining (ROS level detection) is shown in (a), revealing that the ROS level (green fluorescence intensity) was remarkably higher in PC12 cells subjected to OGD (2nd panel) than that in the control group (1st panel). After treatment with 10, 50, or 100 *μ*M GPF (3rd, 4th, and 5th panels, resp.), the fluorescence intensity was lower in PC12 cells than that in cells without GFP treatment (2nd panel), in a dose-dependent manner. The MMP (b) and SOD activity (right panel of (c)) decreased in PC12 cells subjected to OGD (2nd bar) compared to that in the control cells, whereas 10, 50, and 100 *μ*M GPF (3rd, 4th, and 5th panels, resp.) treatment significantly upregulated the OGD-decreased MMP and SOD activity in a dose-dependent manner in PC12 cells. GPF downregulated the OGD-induced upregulation of the MDA level in a dose-dependent manner (left panel of (c)). GPF significantly decreased the OGD-induced upregulation of IL-1*β* (left panels of (d) and (e)) and TNF-*α* (right panels of (d) and (e)) by ELISA (d) and qPCR (e) in PC12 cells. The data of each group were from three independent assays. ^∗^*P* < 0.05 versus 0 mg/kg GFP with OGD; ^∗∗^*P* < 0.01 versus 0 mg/kg GFP with OGD.

Initially, the antioxidative activity of GPF was evaluated in PC12 cells treated with or without OGD using DCFH-DA staining. As shown in Figure 1(a), OGD significantly induced ROS accumulation in PC12 cells, whereas GPF remarkably decreased ROS accumulation induced by OGD in a dose-dependent manner. Moreover, GPF significantly restored the MMP (Figure 1(b)), upregulated SOD activity, and decreased the MDA level in PC12 cells subjected to OGD in a dose-dependent manner (Figure 1(c)).

The anti-inflammatory activity of GPF was determined in the OGD-treated PC12 cells. The proinflammatory factors TNF-*α* and IL-1*β* were examined by ELISA (Figure 1(d)) and qPCR (Figure 1(e)) in the culture medium or in PC12 cells, respectively. The results indicated that GPF clearly reduced the TNF-*α* and IL-1*β* protein levels and mRNA expression induced by OGD in a dose-dependent manner.

### 3.2. GPF Reduced OGD-Induced PC12 Cell Injury

**Figure 2:**
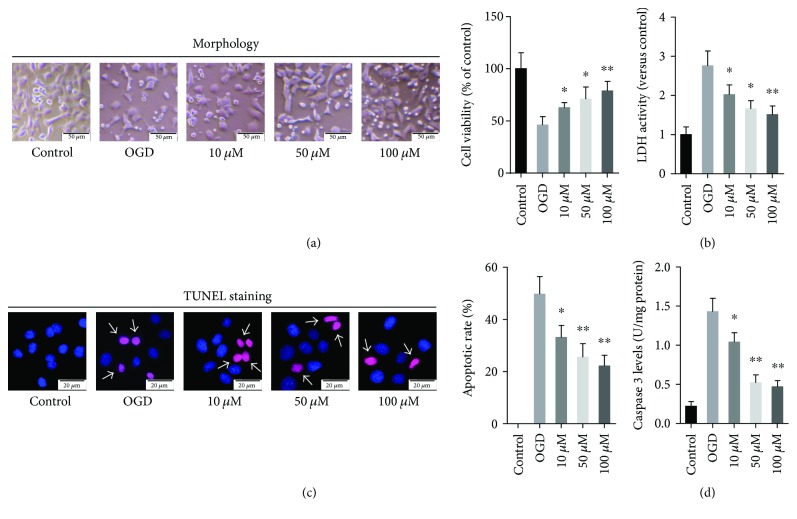
GPF protected the hypoxic-ischemic injury in PC12 cells. To determine the neuroprotective effects of GPF, PC12 cells were pretreated with different doses of GPF (0, 10, 50, or 100 *μ*M, resp.) for 1 h before being subjected to OGD. PC12 cells without GPF and OGD treatment were set as a control. The cellular injury induced by OGD was analyzed by a cellular viability assay (CCK-8 assay) (a) and a LDH activity assay (b). GPF treatment at the indicated doses relieved OGD-induced cellular injury. The amount of apoptotic PC12 cells was determined by TUNEL staining. TUNEL-positive and total (DAPI-positive) number of the cells in each group were counted manually by two independent observers in 6 random microscopic fields (400x), and the percentage of TUNEL-positive cells were subsequently calculated (c). GPF decreased the ratio of TUNEL-positive cells (purple fluorescence, labeled with white arrows) in a dose-dependent manner. Nuclei were stained by DAPI (blue fluorescence). GPF downregulated the OGD-induced levels of caspase-3 in a dose-dependent manner in PC12 cells. The data of each group were from three independent assays. ^∗^*P* < 0.05 versus 0 mg/kg GFP with OGD; ^∗∗^*P* < 0.01 versus 0 mg/kg GFP with OGD.

Next, the effect of GPF on OGD-induced PC12 cell injury was evaluated by the CCK-8 assay which detects the survival cells and the LDH assay which reveals the damaged cells. The results displayed that GPF clearly relieved the cellular injury mediated by OGD in a dose-dependent manner (Figures 2(a) and 2(b)). The TUNEL assay revealed the extent of apoptosis in PC12 cells induced by OGD, while treatment with GPF remarkably decreased the ratio of TUNEL-positive cells (*P* < 0.01; Figure 2(c)). Moreover, GPF significantly reduced the OGD-induced caspase-3 expression in PC12 cells (Figure 2(d)).

### 3.3. GPF Mediates Nrf2 Activation through the PI3K/Akt Signaling Pathway

**Figure 3:**
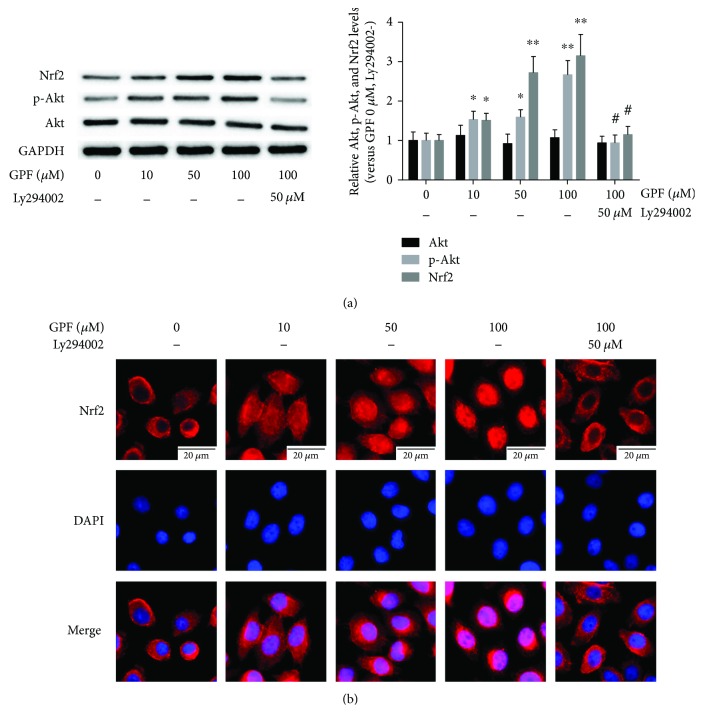
GPF upregulates and activates Nrf2. PC12 cells were treated with different doses of GPF (0, 10, 50, or 100 *μ*M, resp.) for 6 h or treated with PI3K/Akt inhibitor Ly294002 (50 *μ*M) for 1 h followed by treatment with GPF (100 *μ*M) for 6 h. The results of Western blot analysis showed that Nrf2 and p-Akt expression levels were upregulated by 10–100 *μ*M GPF, which were inhibited by pretreatment with the PI3K/Akt inhibitor Ly294002. GPF treatment has no effect on Akt expression in PC12 cells (a). Fluorescence staining showed that 10–100 *μ*M GPF treatment induced Nrf2 (red fluorescence) translocation into the nuclei (blue). Pretreatment with Ly294002 significantly attenuated the GPF-induced nuclear translocation of Nrf2 (b). The data of each group were from three independent assays. ^∗^*P* < 0.05 versus the control group; ^∗∗^*P* < 0.01 versus the control group; ^#^*P* < 0.01 versus the 100 *μ*M GPF group.

Nrf2 plays a pivotal role in regulating redox homeostasis [5]. Thus, we further detected the effect of GPF on Nrf2 expression. The Western blot assay results revealed that both low-dose and high-dose GPF significantly upregulated both Nrf2 and p-Akt expression (*P* < 0.05) but not Akt (*P* > 0.05; Figure 3(a)). Immunofluorescence staining demonstrated that Nrf2 was primarily located in the cytoplasm in normal PC12 cells, yet it was remarkably increased in the nuclei after treatment with GPF, especially with the high-dose treatment. These results suggested that GPF could activate Nrf2 to translocate to the nuclei. Interestingly, after treatment with the PI3K/Akt inhibitor Ly294002, the levels of p-Akt and Nrf2, as well as the Nrf2 nuclear translocation in PC12 cells treated with GPF, were significantly attenuated (Figures 3(a) and 3(b)).

### 3.4. Blocking the PI3K/Akt Pathway or Nrf2 Silencing Reduced the Antioxidative, Anti-Inflammatory, and Neuroprotective Activities of GPF

**Figure 4:**
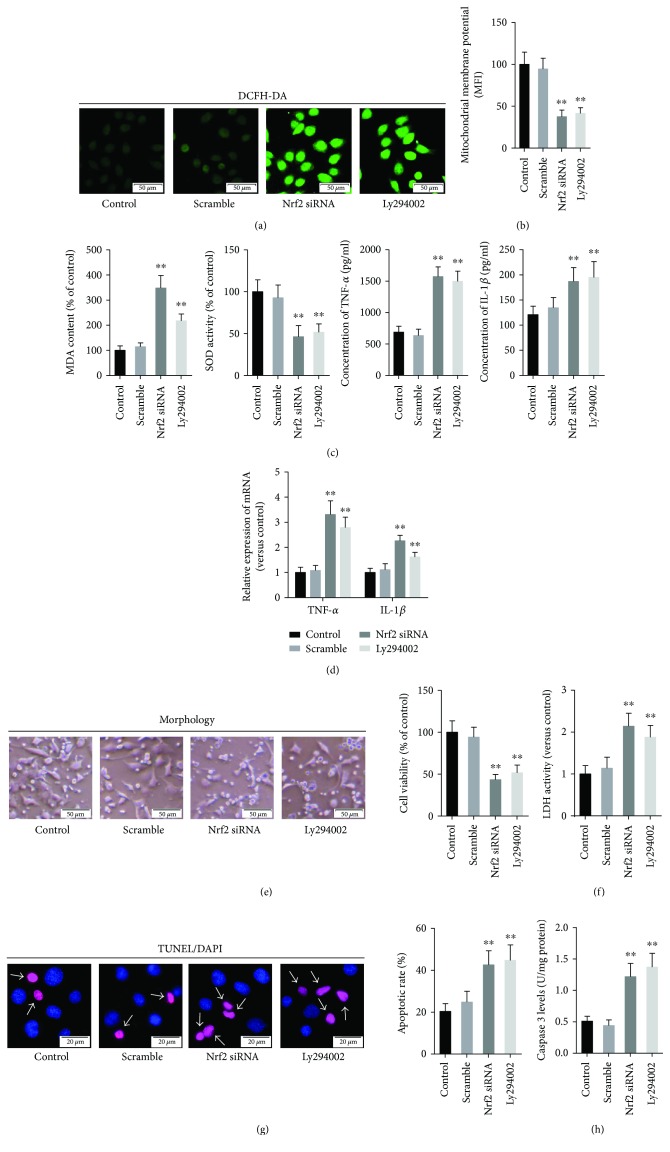
Nrf2 siRNA and Ly294002 attenuated the antioxidative and cell protective activities of GPF. PC12 cells pretreated with Ly294002 (50 *μ*M) for 1 h or transfected with Nrf2 siRNA (50 nM) or scramble RNA was administrated with GPF (100 *μ*M) for 1 h and then subjected to OGD as mentioned above. PC12 cells only treated with GPF and OGD were set as the control group. The ROS level (green fluorescence, in (a)) and MDA level (right panel of (c)) were remarkably increased after Nrf2 siRNA or Ly294002 treatment, compared with the control cells. The MMP (b) and SOD activity (right panel of (c)) were downregulated by Nrf2 siRNA or Ly294002 administration. The mRNA expression (detected by qRT-PCR; upper panel of (d)) in the PC12 cells as well as the protein levels (detected by ELISA; lower panel of (d)) of TNF-*α* and IL-1*β* in the cell culture medium was remarkably upregulated by Nrf2 siRNA or Ly294002 treatment, compared with the control group. The results of the CCK-8 assay and LDH activity detection revealed that Nrf2 siRNA or Ly294002 weakened the protective effects of GPF against OGD (e). Finally, Nrf2 siRNA or Ly294002 administration upregulated the ratio of TUNEL-positive cells and the generation of caspase-3 (f). The data of each group were from three independent assays. ^∗∗^*P* < 0.01 versus the control group.

Continuously, to determine whether PI3K/Akt/Nrf2 pathway is involved in the neuroprotective effects of GPF, PC12 cells were administrated with scramble siRNA, Nrf2 siRNA, or Ly294002 before GPF and OGD treatment, and the oxidative stress, inflammatory response, and cellular injury were subsequently evaluated. The results demonstrated that treatment with scramble siRNA shows no effects on the ROS and MDA levels compared with the control group (PC12 cells only treated with GPF and OGD), whereas treatment with Nrf2 siRNA or Ly294002 significantly increased the ROS (Figure 4(a)) and MDA levels (left panel of Figure 4(c)) (*P* < 0.01, resp.). Compared with the control group, the MMP (Figure 4(b)) and SOD (right panel of Figure 4(c)) activity was significantly decreased in the PC12 cells treated with Nrf2 siRNA or Ly294002 (*P* < 0.01, resp.). The mRNA expression (upper panel of Figure 4(d), by qPCR) in the cells and the protein level (lower panel of Figure 4(d), by ELISA) of TNF-*α* and IL-1*β* in the culture medium were upregulated by Nrf2 siRNA or Ly294002 treatment, compared with those of the control cells (*P* < 0.01, resp.).

Further results indicated that pretreatment with Nrf2 siRNA or Ly294002 significantly reversed the GPF-induced upregulation of the cellular viability rate (Figure 4(e)) and the downregulation of LDH levels (Figure 4(f)) in the PC12 cell induced by OGD. These data indicate that Nrf2 siRNA or Ly294002 diminished the neuroprotective capacity of GPF. In addition, the results showed that treatment with Nrf2 siRNA or Ly294002 increased the percentage of TUNEL-positive cells (Figure 4(g)) and the levels of caspase-3 in PC12 cells (Figure 4(h)).

### 3.5. GPF Lessens Rat Brain Injury in a Rat CIRI Model In Vivo

**Figure 5:**
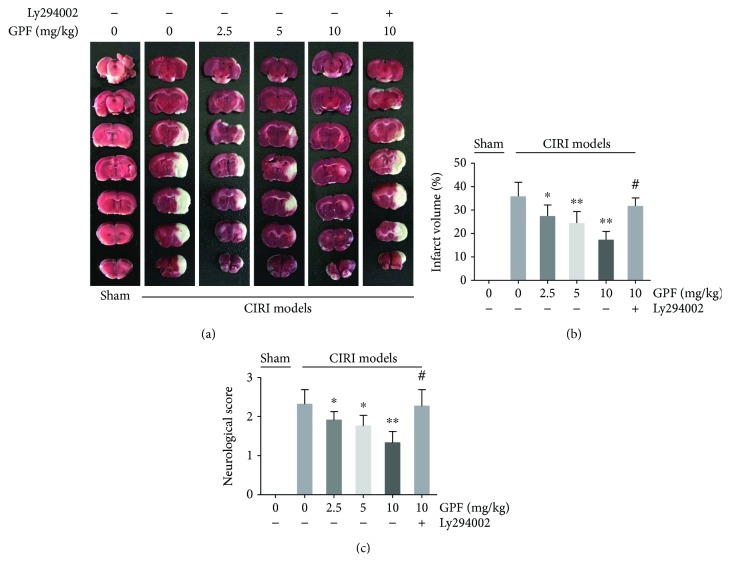
GPF mitigates the cerebral infarction volume and neurological impairment through the PI3K/Akt pathway in a rat CIRI model *in vivo*. Before being subjected to CIRI, rats were pretreated with different dose of GPF (2.5, 5, or 10 mg/kg/day) for 14 days. In some cases, Ly294002 (20 mM, 5 *μ*L) was injected into the lateral ventricle daily for 7 days before CIRI model establishment. The infarct volume was remarkably decreased in the GPF treatment groups, compared with rats without GPF treatment. Yet, the infarct volume further increased after treatment with Ly294002 (a) and (b). The neurological score was reduced in the GPF treatment groups, compared with the CIRI rats without GPF treatment, indicating that GPF could protect nerve function and improve brain function in a dose-dependent manner. Nevertheless, the nerve-protecting and nerve-improving activities of GPF were diminished after treatment with Ly294002 (c). Each group contains at least six rats. ^∗^*P* < 0.05 versus 0 mg/kg GFP in the CIRI models; ^∗∗^*P* < 0.01 versus 0 mg/kg GFP in the CIRI models; ^#^*P* < 0.01 versus 10 mg/kg GFP in the CIRI models.

The neuroprotective activity of GPF was further tested in a rat CIRI model. Wistar rats with or without GPF treatment were sacrificed at 24 h after CIRI, and the infarct volume was evaluated by TTC staining. As shown in Figures 5(a) and 5(b), infarct volume in CIRI rats was remarkably higher than those in the sham group, whereas GPF treatment remarkably reduced the infarction volume in a dose-dependent manner. However, after pretreatment with Ly294002, the infarction volume in the CIRI rats treated with 10 mg/kg GPF further increased (Figures 5(a) and 5(b)).

In the neurological function examination at 24 h after CIRI, the neurological score of the GPF group was significantly lower than that of the CIRI group, in a dose-dependent manner. However, this neuroprotective activity of GPF was diminished after pretreatment with Ly294002 (Figure 5(c)).

### 3.6. GPF Reduces Oxidative Stress in a Rat CIRI Model In Vivo

**Figure 6:**
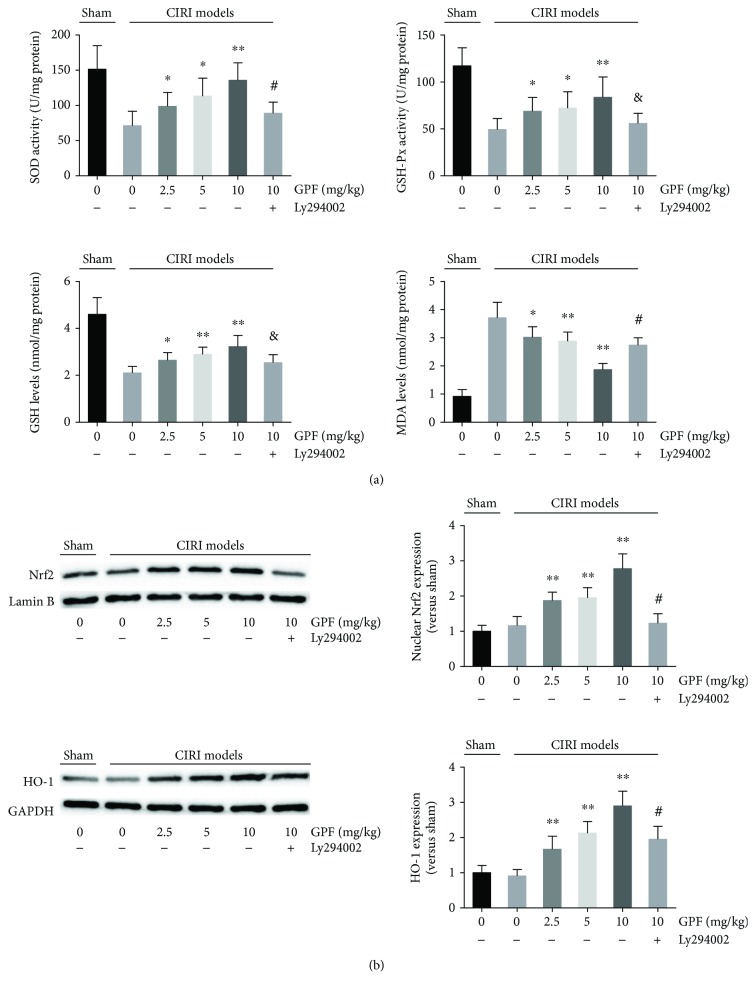
GPF decreases oxidative stress through the PI3K/Akt pathway in a rat CIRI model. The SOD, GSH, and GSH-Px activities as well as the MDA level were analyzed in the indicated groups in the brain tissue of MCAO rats (a). (b) Nrf2 nuclear translocation was determined by a Western blot assay in the isolated nuclear fraction of the brain tissue of MCAO rats in each group (upper left panels of (b)). Densitometric analysis of the Nrf2 Western blot images is presented in the upper right panels of (b). The HO-1 expression and the corresponding densitometry of the indicated groups were analyzed as well (lower panels of (b)). Each group contains at least six rats. ^∗^*P* < 0.05 versus 0 mg/kg GPF in the CIRI models; ^∗∗^*P* < 0.01 versus 0 mg/kg GPF in the CIRI models; ^&^*P* < 0.01 versus 10 mg/kg in the CIRI models; ^#^*P* < 0.01 versus 10 mg/kg in the CIRI models.

The antioxidant capacity of GPF was then examined in vivo. The MDA levels in brain tissue in CIRI rats were remarkably higher than those in the sham group. In contrast, SOD and GSH-Px activities, as well as the GSH levels, in brain tissue in CIRI rats were significantly lower than those in the sham rats. Interestingly, intraperitoneal injection with GPF daily for 14 days could significantly diminish the MDA levels as well as increase the GSH levels, SOD, and GSH-Px activities in a dose-dependent manner. Ly294002 administration significantly weakened the ability of GPF to attenuate oxidative stress (Figure 6(a)). Simultaneously, a Western blot assay was employed to evaluate the translocation of Nrf2 and the levels of HO-1, an oxidation-redox stress regulation protein. The results showed that 10–100 *μ*M GPF could increase Nrf2 expression in the nuclei and upregulate the HO-1 protein levels in the rat brain tissue. However, Ly294002 significantly decreased the GPF-induced Nrf2 nuclear translocation and HO-1 expression in the rat brain tissue (Figure 6(b)).

### 3.7. GPF Mitigates the Brain Inflammatory Response in a Rat CIRI Model In Vivo

**Figure 7:**
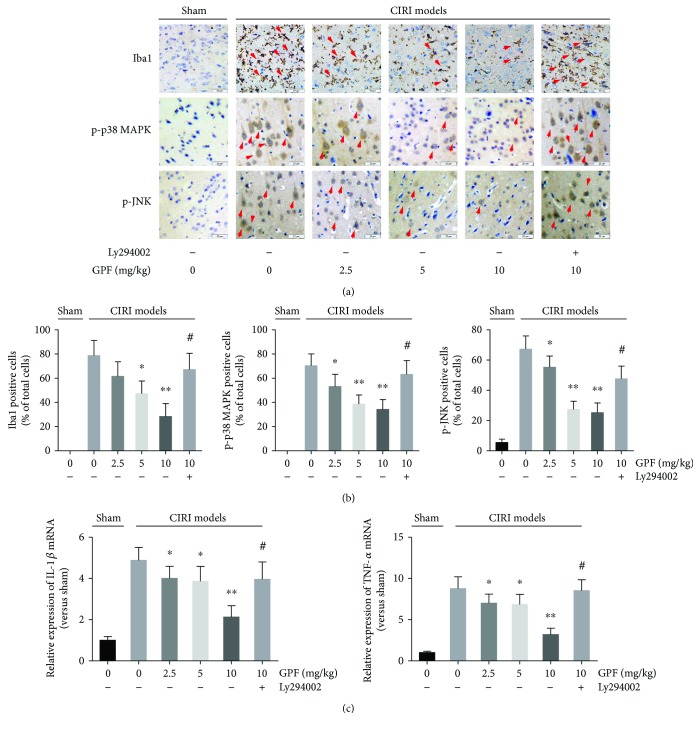
GPF diminished the inflammatory reaction through the PI3K/Akt pathway in a rat CIRI model *in vivo*. Iba1, phosphorylated p38 (p-p38), and phosphorylated JNK (p-JNK) were immunohistochemically stained in the rat brain slides of the indicated groups, respectively (a). The number of Iba1, p-p38, and p-JNK positively stained cells was counted (b). The gene expression levels of IL-1*β* and TNF-*α* in the indicated groups were detected by qPCR (c). Each group contains at least six rats. ^∗^*P* < 0.05 versus 0 mg/kg GPF in the CIRI models; ^∗∗^*P* < 0.01 versus 0 mg/kg GPF in the CIRI models; ^#^*P* < 0.01 versus 10 mg/kg GPF in the CIRI models.

Next, the anti-inflammatory activity of GPF was detected in a rat CIRI model. The number of Iba1-, p-p38-, and p-JNK-positive cells as well as IL-1*β* and TNF-*α* expression in the rat brain of the CIRI model was significantly higher than those in the sham control rats. Intraperitoneal injection with GPF daily for 14 days could significantly decrease the CIRI-induced increase in the number of Iba1-, p-p38-, and p-JNK-positive cells as well as the expression of IL-1*β* and TNF-*α*, in a dose-dependent manner. However, the GPF-reduced inflammatory reaction was impaired in CIRI rats after Ly294002 administration (Figure 7).

### 3.8. GPF Relieved Cell Apoptosis in a Rat CIRI Model In Vivo

**Figure 8:**
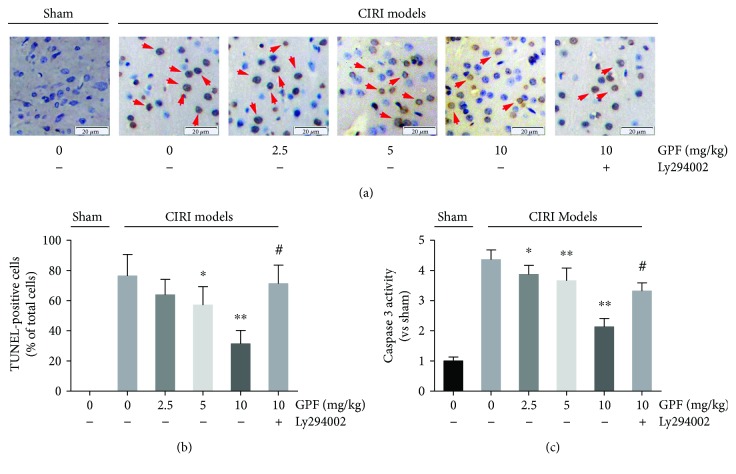
GPF relieved cell apoptosis in a rat CIRI model *in vivo*. Rat brain slices of the indicated groups were stained with a TUNEL assay kit (a). The TUNEL-positive cells (yellow-brown, elliptical, or round) of each corresponding group were counted (b). The activity of caspase-3 was detected in the rat brain samples of the indicated groups (c). Each group contains at least six rats. ^∗^*P* < 0.05 versus 0 mg/kg GPF in the CIRI models; ^∗∗^*P* < 0.01 versus 0 mg/kg GPF in the CIRI models; ^#^*P* < 0.01 versus 10 mg/kg GPF in the CIRI models.

The antiapoptotic activity of GPF was then tested in an *in vivo* system using a rat CIRI model. The TUNEL-stained cells were yellow-brown, elliptical, or round. Compared with the sham group, in which TUNEL-positive cells were not observed, numerous TUNEL-positive cells were visible in the rat brain tissue of the positive control (CIRI) group. GPF, in the range of 10 to 100 *μ*M, significantly reduced the number of TUNEL-stained cells in a dose-dependent manner (*P* < 0.05). Yet, the percentage of the TUNEL-positive cells was significantly reversed after pretreatment with Ly294002 (Figures 8(a) and 8(b)). In addition, by Western blot analysis, caspase-3 expression in the rat brain was upregulated by CIRI and decreased by GPF administration (10–100 *μ*M). As expected, pretreatment with Ly294002 significantly attenuated the CIRI-induced caspase-3 expression (Figure 8(c)).

## 4. Discussion

Oxidative stress is widely involved in many pathophysiological processes such as aging, inflammation, and tumorigenesis [20]. A wealth of evidence suggests that oxidative stress induced by the overproduction of ROS is the core mechanism of CIRI occurrence and development. Therefore, reducing oxidative stress is believed to be the key mechanism to treat ischemic stroke [3, 4]. GPF has been shown to resist DNA, lipid, and protein damage induced by oxidative stress [15, 16, 21]. Here, we demonstrated that GPF could reduce the ROS level in the PC12 cell OGD model as well as diminish the MMP damage induced by OGD (Figure 1). Furthermore, GPF administration could upregulate SOD activity and downregulate the MDA level in PC12 cells (Figure 1). In addition, it could increase GSH, SOD, and GSH-Px expression (Figure 6) but decrease the MDA level (Figure 1) in the brain tissue of CIRI rats. These findings suggest that GPF could mitigate oxidative stress in nerve cells after CIRI.

The inflammatory reaction of the nervous system is another important pathological characteristic in ischemic cerebrovascular disease. After CIRI, ROS activate inflammatory signaling pathways either directly or indirectly through oxidative products, such as nuclear factor-*κ*B and MAPK pathways, contribute to the proliferation and activation of microglia, and release inflammatory factors, such as TNF-*α* and IL-1*β* [22–25]. Inflammation, in turn, contributes to the formation of ROS. Both pathways form a positive feedback loop, thus aggravating the damage to the nerve tissue [24]. We found that GPF significantly decreased the synthesis and release of TNF-*α* and IL-1*β* in a PC12 cell OGD model *in vitro* (Figure 1) and a rat CIRI model *in vivo* (Figure 4). Microglia are the major immune cells in the central nervous system. Following CIRI, proliferated microglia activate and release inflammatory factors and ROS [26]. Herein, we demonstrated that GPF noticeably diminishes the number of microglia in the ischemia/reperfusion region in a dose-dependent manner. In addition, GPF clearly decreased the numbers of phosphorylated p38 MAPK- and JNK-positive cells after CIRI. p38 MAPK and JNK are two important members of the MAPK family, both of which play a key regulatory role on inflammatory reactions and can be activated after CIRI [27]. Our findings indicated that the anti-inflammatory effects of GPF may occur through the p38 MAPK and JNK pathways in the rat CIRI model. In addition, GPF also revealed antiapoptotic activity, which attenuates the oxidative stress- and inflammation-induced PC12 cell apoptosis *in vitro* (Figure 2) as well as rat brain neuron cell apoptosis in a CIRI/MCAO model *in vivo* (Figure 4).

The precise mechanism of the antioxidative activity of GPF still remains poorly understood so far. Gallic acid and paeoniflorin, the two major components of GPF, have been reported to alleviate oxidative stress through Nrf2 activation [28, 29]. Nrf2 knockout mice are sensitive to CIRI [9, 10], but an Nrf2 agonist can mitigate CIRI [10–13]. Therefore, this study further detected whether GPF could activate the Nrf2 signaling pathway. GPF stimulation induced Nrf2 protein expression in PC12 cells *in vitro* (Figure 3) and promoted Nrf2 and HO-1 expression in the brain tissue of CIRI rats *in vivo* (Figure 6). HO-1 is a rate-limiting enzyme in the process of heme catabolism [30]. There is an antioxidant response element in its promoter region, which is regulated by Nrf2 [30]. HO-1 directly affects the antioxidative balance in the body, and it has anti-inflammatory and antiapoptotic activities as well [31, 32]. Moreover, GPF administration not only induced Nrf2 expression but also promoted Nrf2 transfer from the cytoplasm to the nucleus in PC12 cells, in a dose-dependent manner (Figure 3). Nrf2 knockdown by siRNA attenuated the cellular protective, antioxidative, and anti-inflammatory properties of GPF in PC12 cells (Figures 1, 2, and 4). Together, these findings suggest that GPF may regulate antioxidant response element-related gene expression through promoting Nrf2 expression and activation to play antioxidative and anti-inflammatory effects after CIRI.

Under conditions of oxidative stress or electrophilic stimulation, the structure of Keap1 is changed, resulting in its disassociation from Nrf2, thus activating Nrf2. In addition, PI3K/Akt [33], MAPK [34], and PKC [35] can phosphorylate the serine site of Nrf protein to weaken its binding with Keap1, leading to Nrf2 activation under oxidative stress or electrophilic stimulation, thereby enhancing its expression level and promoting its translocation to the nucleus. As an important component of GPF, paeoniflorin has been reported to activate the PI3K/Akt signaling pathway [36]. In this study, we determined whether GPF could mediate Nrf2 activation through activating the PI3K/Akt pathway (Figure 3). Our results showed that GPF administration elevated the p-PI3K and p-Akt levels in PC12 cells in a dose-dependent manner. Importantly, the ability to promote Nrf2 expression and nuclear translocation of GPF was weakened after administration of the PI3K inhibitor Ly294002 (Figure 3). A further study *in vitro* demonstrated that Ly294002 administration could inhibit the effects of GPF on oxidative stress and inflammatory factor levels in PC12 cells after OGD as well as weaken the protective effect of GPF on nerve cells (Figure 4). In addition, Ly294002 treatment inhibited the promoting effect of GPF on the expression of Nrf2/HO-1 protein in the brain tissue of CIRI rats, suppressed the antioxidative stress and inflammatory activities of GPF, and aggravated nerve injury after CIRI. These data demonstrated that GPF phosphorylated and activated Nrf2 through activating the PI3K/Akt signaling pathway to perform its antioxidative, anti-inflammatory, and antiapoptotic activities as well as neuroprotective effects.

## 5. Conclusion

GPF mitigated oxidative stress and inflammatory reactions after CIRI as well as played a neuroprotective effect both *in vitro* and *in vivo*. GPF administration upregulated Nrf2 expression and promoted Nrf2 nuclear translocation in PC12 cells through activating the PI3K/Akt pathway. In addition, Nrf2 knockdown or PI3k/Akt inhibition with Ly294002 blocked the protective effect of GPF on PC12 cells after OGD. Moreover, GPF could antagonize the CIRI-induced neurotoxicity; thus, it could be a potential therapeutic agent for cerebral infarction.

## Abbreviations

CIRI: Cerebral ischemia-reperfusion injury
ELISA: Enzyme-linked immunosorbent assay
GPF: 6′-*O*-galloylpaeoniflorin
HO-1: Heme oxygenase-1
Iba1: Ionized calcium-binding adapter molecule 1
IL-1*β*: Interlukin-1*β*
Keap1: Kelch-like ECH-associated protein 1
LDH: Lactate dehydrogenase
MCAO: Middle cerebral artery occlusion
MKP1: Mitogen-activated protein kinase phosphatase 1
MMP: Mitochondrial membrane potential
Nrf2: Nuclear factor-erythroid 2-related factor 2
OGD: Oxygen-glucose deprivation
p-JNK: Phosphorylated c-Jun N-terminal kinase
PI3K: Phosphoinositide 3-kinase
qPCR: Real-time quantitative polymerase chain reaction
ROS: Reactive oxygen species
SOD: Superoxide dismutase
MDA: Malondialdehyde
TNF-*α*: Tumor necrosis factor alpha
TTC: 2,3,5-triphenyl tetrazolium chloride.
